# Genetic and immune crosstalk between severe burns and blunt trauma: A study of transcriptomic data

**DOI:** 10.3389/fgene.2022.1038222

**Published:** 2022-09-30

**Authors:** Xiaoming Chen, Kuan Wang, Dazhuang Li, Mingyue Zhao, Biao Huang, Wenxing Su, Daojiang Yu

**Affiliations:** ^1^ Department of Plastic and burns Surgery, The Second Affiliated Hospital of Chengdu Medical College (China National Nuclear Corporation 416 Hospital), Chengdu, China; ^2^ Department of Cosmetic Plastic and burns Surgery, The First Affiliated Hospital of Chengdu Medical College, Chengdu, China; ^3^ Department of Orthopedics, The Affiliated Hospital of Yangzhou University, Yangzhou University, Yangzhou, China; ^4^ Department of Periodontology, Affiliated Stomatological Hospital of Zunyi MedicalUniversity, Zunyi, China

**Keywords:** burns, blunt trauma, bioinformatics, differentially expressed genes, immune cell infiltration, core immune-related genes

## Abstract

**Background:** Severe burns and blunt trauma can lead to multiple organ dysfunction syndrome, the leading cause of death in intensive care units. In addition to infection, the degree of immune inflammatory response also affects prognosis. However, the characteristics and clinical relevance of the common mechanisms of these major diseases are still underexplored.

**Methods:** In the present study, we performed microarray data analysis to identify immune-related differentially expressed genes (DEGs) involved in both disease progression in burns and blunt trauma. Six analyses were subsequently performed, including gene enrichment analysis, protein‐protein interaction (PPI) network construction, immune cell infiltration analysis, core gene identification, co-expression network analysis, and clinical correlation analysis.

**Results:** A total of 117 common immune-related DEGs was selected for subsequent analyses. Functional analysis emphasizes the important role of Th17 cell differentiation, Th1 and Th2 cell differentiation, Cytokine-cytokine receptor interaction and T cell receptor signaling pathway in these two diseases. Finally, eight core DEGs were identified using cytoHubba, including CD8A, IL10, CCL5, CD28, LCK, CCL4, IL2RB, and STAT1. The correlation analysis showed that the identified core DEGs were more or less significantly associated with simultaneous dysregulation of immune cells in blunt trauma and sepsis patients. Of these, the downregulation of CD8A and CD28 had a worse prognosis.

**Conclusion:** Our analysis lays the groundwork for future studies to elucidate molecular mechanisms shared in burns and blunt trauma. The functional roles of identified core immune-related DEGs and dysregulated immune cell subsets warrant further in-depth study.

## Introduction

Burns caused by physical or chemical factors are characterized by high morbidity and mortality, causing unpredictable and severe injuries to patients. Thermal injuries caused by hot liquids, solids, or fire account for a large proportion of burns injuries (Lee, 1997). According to a 2018 report by the World Health Organization, there are about 11 million burns patients worldwide each year, and although the mortality rate has dropped from the 300,000 recorded in 2011 [(Peck, 2011)], the death toll is still as high as 180,000 [(Pereira et al., 2013)]. The improvement of the survival rate of burns patients is mainly attributed to the improvement of intensive care level, the improvement of wound care, and the effective control of infection (Cioffi et al., 1993; Finnerty et al., 2007). Most patients with severe burns require prompt and specialized burns care to reduce morbidity and mortality. Factors leading to high mortality in severe burns include hypovolemic shock, immunosuppression (Finnerty et al., 2007; Xiao et al., 2011), excessive inflammation (Stanojcic et al., 2018), and hypermetabolic response (Jeschke et al., 2011), and severe burns can also lead to severe infection, sepsis, and multiple organ function disorder syndrome (MODS), which can also lead to death in severely burnsed patients (Martin et al., 2003).

In the early stage of blunt traumatic injury, the skin and mucosal barriers and cell membrane microcarriers are generally destroyed, resulting in the release of various pathogenic factors and the activation of innate immune responses (Lord et al., 2014). These processes can help blunt traumatized patients quickly escape from the dangerous period, but may also cause serious complications disease or even death (Callcut et al., 2016; Gabbe et al., 2017; Mira et al., 2017; Sauaia et al., 2017). After severe blunt trauma in the head, chest, abdomen and other parts, the innate immune system is activated, and a series of complex and heterogeneous multisystem reactions occur (Keel and Trentz, 2005; Adib-Conquy and Cavaillon, 2009; Minei et al., 2012; Cabrera et al., 2017; Dijkink et al., 2018). For example, patients with blunt abdominal blunt trauma who underwent splenectomy had decreased T cell responses to hemagglutinin, decreased lymphocyte numbers, decreased IgM levels, and no significant changes in the expression levels of C3, C4, and C5 [(Downey et al., 1987)]. However, CD46 expression was significantly reduced 48 h after blunt trauma, with or without splenectomy (Amara et al., 2010).

Deaths from severe burns and blunt trauma occur frequently in intensive care units. In addition, severe burns are inherently a special type of trauma. From a physiological point of view, these two diseases are the result of damage to the normal physiological functions of the body due to various external factors. In the course of treatment, the exploration of biomarkers for various types of major disease progression and prognosis is necessary. The purpose of this study to combine the two diseases is to explore the genetic and immune crosstalk between major diseases caused by different etiologies.

In this study, we analyzed the microarray data to identify common functional genes involved in immune regulation under different injury conditions, and initially revealed the underlying molecular mechanism. We found that a variety of immune-related biological functions are dysfunctional after blunt trauma and burns. We then identified common immune-related genes, analyzed the proportion of immune cells, and explored their common relationships. Finally, we evaluated the diagnostic and prognostic value of common immune-related genes to determine their potential research significance and clinical application as novel biomarkers.

## Materials and methods

### Raw data collection

The gene expression profile of GSE11375 (Warren et al., 2009) and GSE77791 [(Tabone et al., 2018)] were downloaded from the Gene Expression Omnibus (GEO) database (http://www.ncbi.nlm.nih.gov/geo) (Edgar et al., 2002), which is a public database containing a large number of high-throughput sequencing and microarray data sets submitted by research institutes worldwide. The two datasets were based on the GPL570 platform (Affymetrix Human Genome U133 Plus 2.0 Array). The GSE11375 dataset contains 158 adult patients with severe blunt trauma and 26 healthy volunteers. The GSE77791 dataset contains 30 samples from severely burnsed patients with a total burns surface area (TBSA) range from 30 to 98 and 13 samples from healthy volunteers. Inclusion/exclusion criteria, clinical descriptions, and ethics for the severe blunt trauma (Warren et al., 2009) and burns (Venet et al., 2015; Tabone et al., 2018) cohort have been previously published elsewhere. Because this study is a secondary analysis of transcriptome data from previously published public databases, the rationale for its sample size and statistical analysis of baseline data have been confirmed in previous studies.

### Data preprocessing and integration

GEO2R (Barrett et al., 2013) (www.ncbi.nlm.nih.gov/geo/ge2r) is an online analysis tool developed based on 2 R packages (GEOquery and Limma). The GEOquery package is used to read data, and the Limma package is used to calculate the differential expression multiple. We used GEO2R to compare gene expression profiles between different groups to determine the DEGs between the diseased group and the control group. Probe sets with no corresponding gene symbols or genes with more than one probe set were removed or averaged, respectively. “*p* < 0.01 and |logFC| ≥ 1” were defined as the thresholds for the screening of differentially expressed genes (DEGs). A list of immune-related genes was downloaded from the Immunology Database and Analysis Portal database (ImmPort; https://www.immport.org). Finally, Venn diagrams were intersected to obtain common immune-related DEGs for blunt trauma and burns.

### Enrichment analyses of immune-related DEGs

In order to better understand the main biological functions of immune-related DEGs for blunt trauma and burns, we analyzed the Gene Ontology (GO) and Kyoto Encyclopedia of Genes and Genomes (KEGG) pathways *via* KOBAS 3.0 database (Bu et al., 2021), a Web server for gene/protein functional annotation and functional enrichment developed by Peking University, which collects 4,325 species functional annotation information. Adjusted *p*-value < 0.05 was considered significant.

### PPI network construction and analysis of core immune-related DEGs

Search Tool for the Retrieval of Interacting Genes (STRING; http://string-db.org) (version 11.5) (Franceschini et al., 2013) can search for the relationship between proteins of interest, such as direct binding relationships, or coexisting upstream and downstream regulatory pathways, to construct a PPI network with complex regulatory relationships. Interactions with a combined score over 0.4 were considered statistically significant. Cytoscape (http://www.cytoscape.org) (version 3.9.0) (Smoot et al., 2011) was used to visualize this PPI network. The core immune-related DEGs were identified by using the cytoHubba plug-in of Cytoscape. Here, we used six common algorithms (Stress, MNC, Degree, Closeness, Radiality, EPC) to evaluate and select core immune-related DEGs. Subsequently, we constructed a co-expression network of these genes *via* GeneMANIA (http://www.genemania.org/) (Warde-Farley et al., 2010), which is a reliable tool for identifying internal associations in gene sets.

### Immune infiltration analysis

The Immune Cell Abundance Identifier (ImmuCellAI) (Miao et al., 2020) (http://bioinfo.life.hust.edu.cn/ImmuCellAI/#!/) provides comprehensive predictions of immune cell abundance by assessing the abundance of 24 immune cell types in gene expression datasets including RNA-Seq and microarray data, of which 24 The cells consist of 18 T cell subtypes and 6 other immune cells: B cells, NK cells, monocytes, macrophages, neutrophils, and DC cells. The sum of all percentages of 24 infiltrating immune cells was defined as the infiltration score. *t*-test was used to compare the differential immune cells between the disease group and the control group. Spearman correlation analysis was used to explore the correlation between immune cells and core immune-related DEGs.

### Clinical relevance of core immune-related DEGs in blunt trauma and burns

To verify our results, the expression of core immune-related DEGs was extracted from GSE36809 (Xiao et al., 2011) and GSE19743 (Zhou et al., 2010), and the difference between disease group and normal group was analyzed by *t* test. To verify the diagnostic value of core immune-related DEGs, we performed receiver operating characteristic curve analysis. Since there is survival information for the burns cohort in the GSE19743 dataset, we compared the differences in core immune-related DEGs between the survival and non-survival groups.

### Statistical analysis

All statistical analyses were performed using R version 4.0.2. *p*-value < 0.05 was considered statistically significant. Volcano plots, bubble plots, histograms and ROC plots were drawn by the R package “ggplot2”. Corrplot package was used to draw a correlation heatmap to visualize the correlation of 24 types of infiltrating immune cells.

## Results

### Identification of immune-related DEGs

**FIGURE 1:**
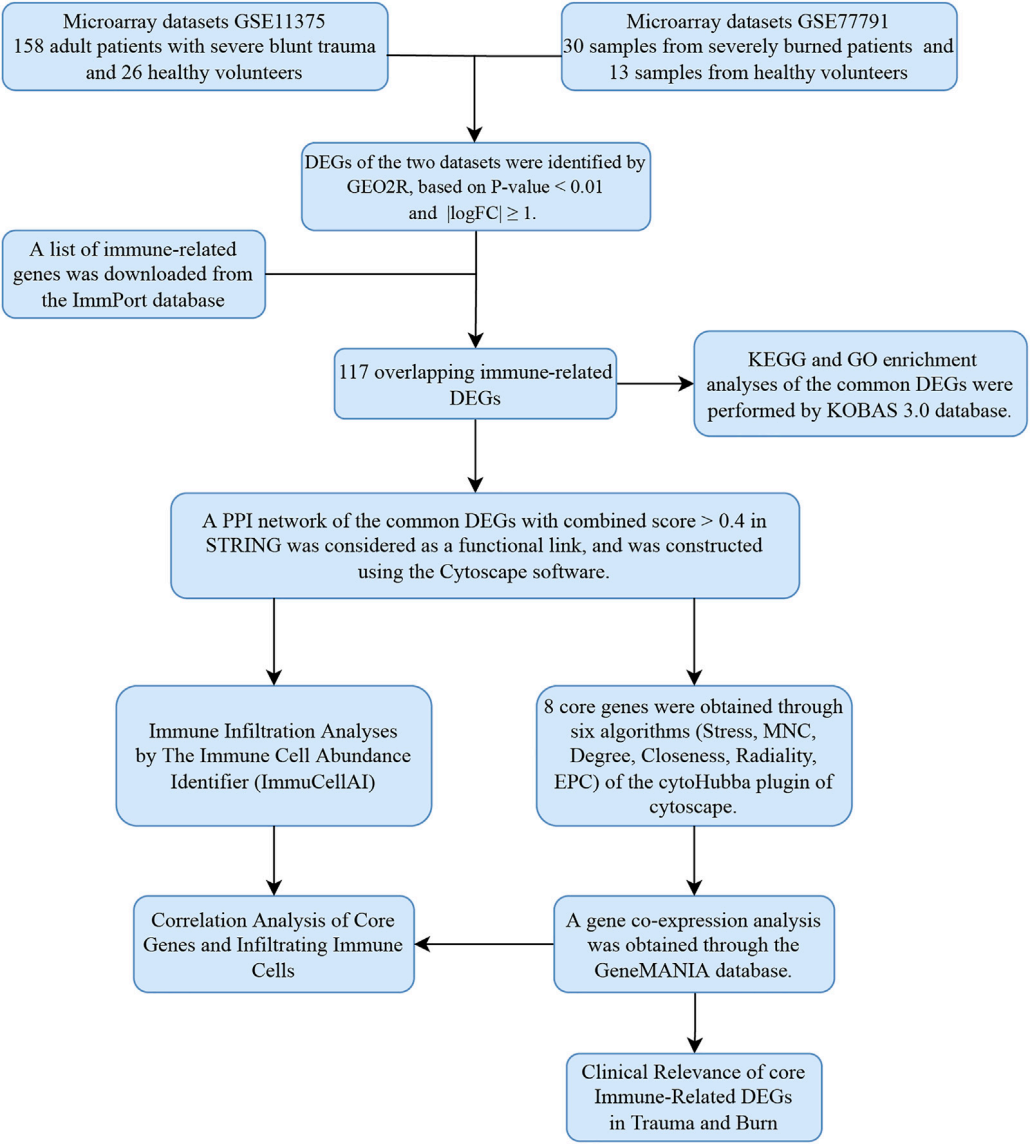
Research design flow chart.

**FIGURE 2:**
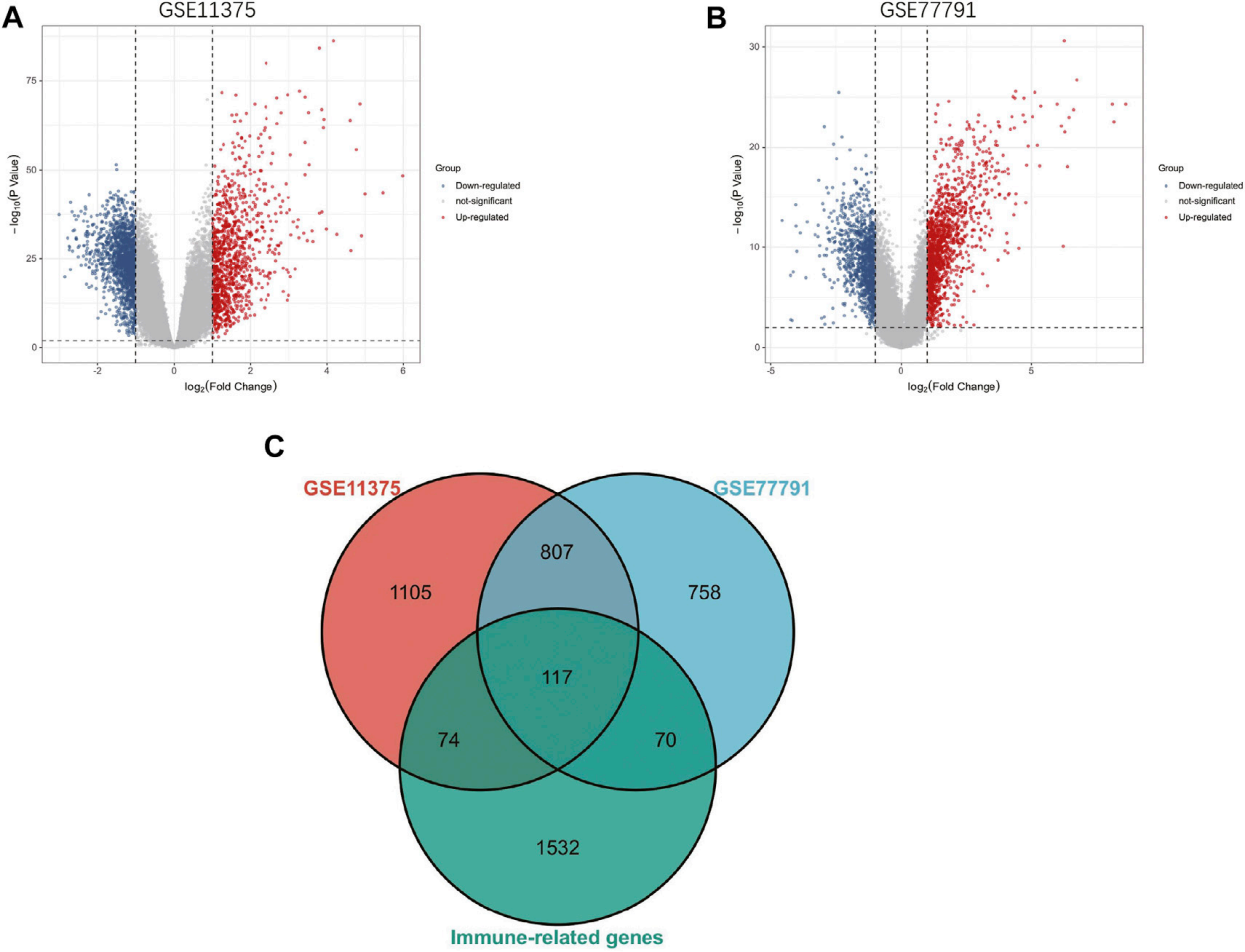
**(A)** The volcano map of GSE11375. **(B)** The volcano map of GSE77791. **(C)** Venn diagram show that 117 overlapping immune-related DEGs in GSE11375, GSE77791 and ImmuCellAI database.

The research flowchart of this research was shown in Figure 1. After standardizing the microarray results, 2103 DEGs and 1752 DEGs were identified in GSE11375 and GSE77791 (Figures 2A,B). Through Venn diagram calculation, we obtained 117 overlapping immune-related DEGs in GSE11375, GSE77791 and ImmPort database (Figure 2C and Supplementary Table S1).

### Analysis of the functional characteristics

**FIGURE 3:**
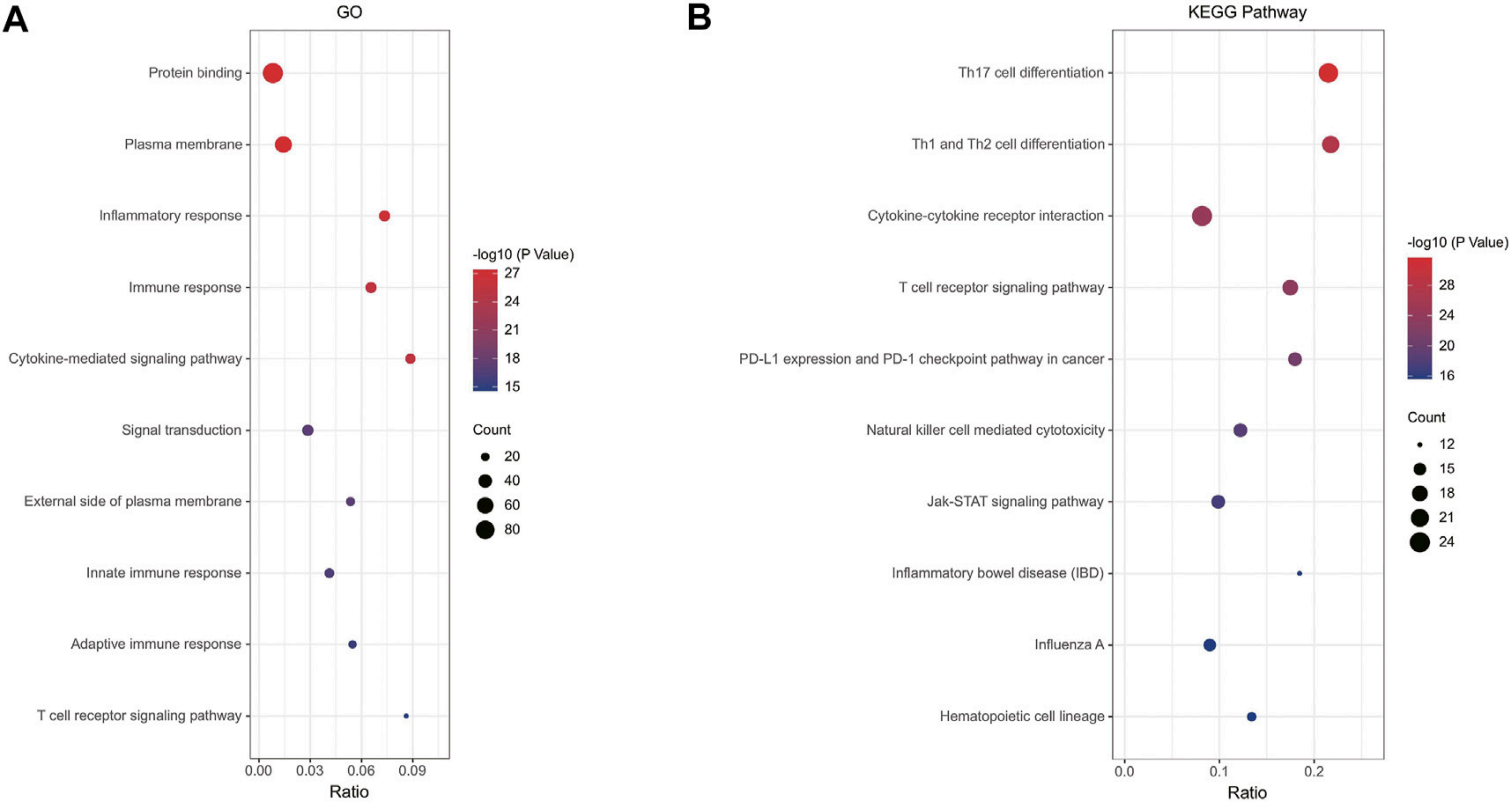
**(A)** Enrichment result of overlapping immune-related DEGs GO term; **(B)** Enrichment result of overlapping immune-related DEGs KEGG pathway. Adjusted *p*-value < 0.05 was considered significant.

In order to analyze the biological functions and pathways involved in immune-related DEGs, GO and KEGG Pathway enrichment analysis were performed. GO analysis results show that immune-related DEGs were significantly enriched in protein binding, plasma membrane, inflammatory response, immune response and cytokine-mediated signaling pathway (Figure 3A). KEGG pathway analysis showed that the DEGs were mainly concentrated in Th17 cell differentiation, Th1 and Th2 cell differentiation, Cytokine-cytokine receptor interaction, T cell receptor signaling pathway and PD-L1 expression and PD-1 checkpoint pathway in cancer (Figure 3B).

### PPI network construction and analysis of core immune-related DEGs

**FIGURE 4:**
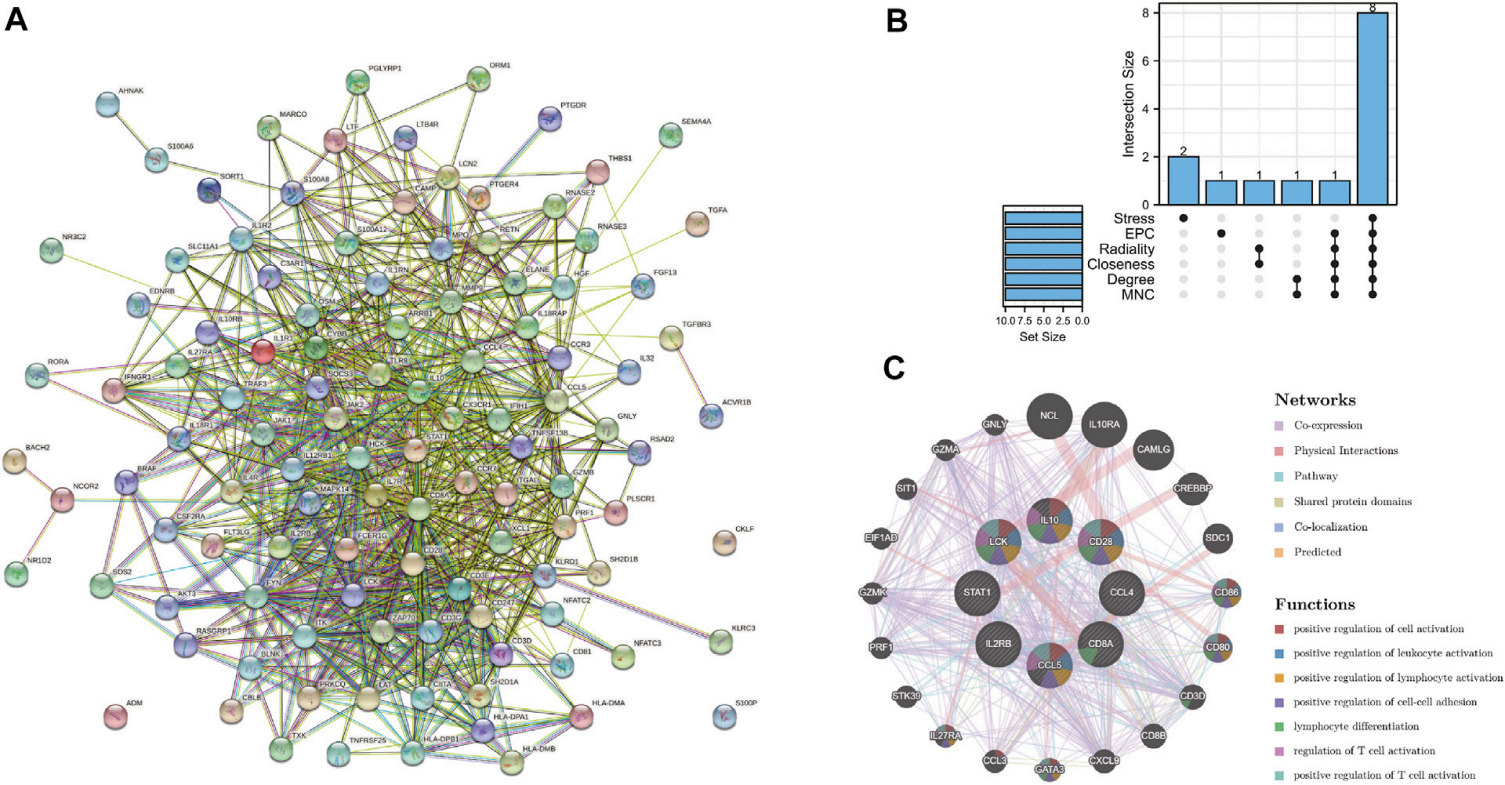
**(A)** PPI network constructed using the STRING database. **(B)** The Venn diagram showed that six algorithms have screened out 8 core immune-related DEGs. **(C)** Core immune-related DEGs and their co-expression genes were analyzed *via* GeneMANIA.

The PPI network of the immune-related DEGs with combined scores greater than 0.4 was constructed using Cytoscape, which contained 112 nodes and 814 interaction pairs (Figure 4A). Through the six algorithms of plug-in cytoHubba, we have calculated the top 10 core genes (Table 1). After taking the intersection of the Venn diagrams, we found 8 overlapping core genes, including CD8A, IL10, CCL5, CD28, LCK, CCL4, IL2RB, and STAT1 (Figure 4B). Table 2 shows their full names and related functions. Based on the GeneMANIA database, we analyzed the co-expression network and related functions of these genes. These genes showed the complex PPI network with the co-expression of 65.17%, physical interactions of 19.21%, pathway of 7.87%, shared protein domains of 3.03%, co-localization of 2.89% and predicted of 1.82% (Figure 4C). These genes are mainly involved in the activation of leukocyte, lymphocytes, etc.

**TABLE 1:** The top 10 core immune-related DEGs rank in cytoHubba.

Stress	MNC	Degree	Closeness	Radiality	EPC
CD8A	CD8A	CD8A	CD8A	IL10	CD8A
IL10	IL10	IL10	IL10	CD8A	IL10
MAPK14	CCL5	CCL5	CCL5	CCL5	CCL5
CCL5	CD28	CD28	CCL4	CCL4	IL7R
LCK	LCK	LCK	CD28	STAT1	CD28
ARRB1	CCL4	CCL4	LCK	IL2RB	LCK
CD28	IL2RB	IL2RB	STAT1	LCK	IL2RB
STAT1	IL7R	IL7R	IL2RB	MMP9	STAT1
CCL4	STAT1	STAT1	IL7R	CD28	CCL4
IL2RB	ZAP70	ZAP70	MMP9	IL7R	CCR7

**TABLE 2:** The details of the core immune-related DEGs.

No.	Gene symbol	Full name	Function
1	CD8A	CD8a Molecule	The CD8 antigen is a cell surface glycoprotein found on most cytotoxic T lymphocytes that mediates efficient cell-cell interactions within the immune system
2	IL10	Interleukin 10	The protein encoded by this gene is a cytokine produced primarily by monocytes and to a lesser extent by lymphocytes. This cytokine has pleiotropic effects in immunoregulation and inflammation
3	CCL5	C-C Motif Chemokine Ligand 5	This gene is one of several chemokine genes clustered on the q-arm of chromosome 17. Chemokines form a superfamily of secreted proteins involved in immunoregulatory and inflammatory processes
4	CD28	CD28 Molecule	The protein encoded by this gene is essential for T-cell proliferation and survival, cytokine production, and T-helper type-2 development
5	LCK	LCK Proto-Oncogene, Src Family Tyrosine Kinase	This gene is a member of the Src family of protein tyrosine kinases (PTKs). The encoded protein is a key signaling molecule in the selection and maturation of developing T-cells
6	CCL4	C-C Motif Chemokine Ligand 4	The protein encoded by this gene is a mitogen-inducible monokine and is one of the major HIV-suppressive factors produced by CD8^+^ T-cells
7	IL2RB	Interleukin 2 Receptor Subunit Beta	The interleukin 2 receptor, which is involved in T cell-mediated immune responses, is present in 3 forms with respect to ability to bind interleukin 2
8	STAT1	Signal Transducer And Activator Of Transcription 1	In response to cytokines and growth factors, STAT family members are phosphorylated by the receptor associated kinases, and then form homo- or heterodimers that translocate to the cell nucleus where they act as transcription activators

### Immune infiltration analysis

**FIGURE 5:**
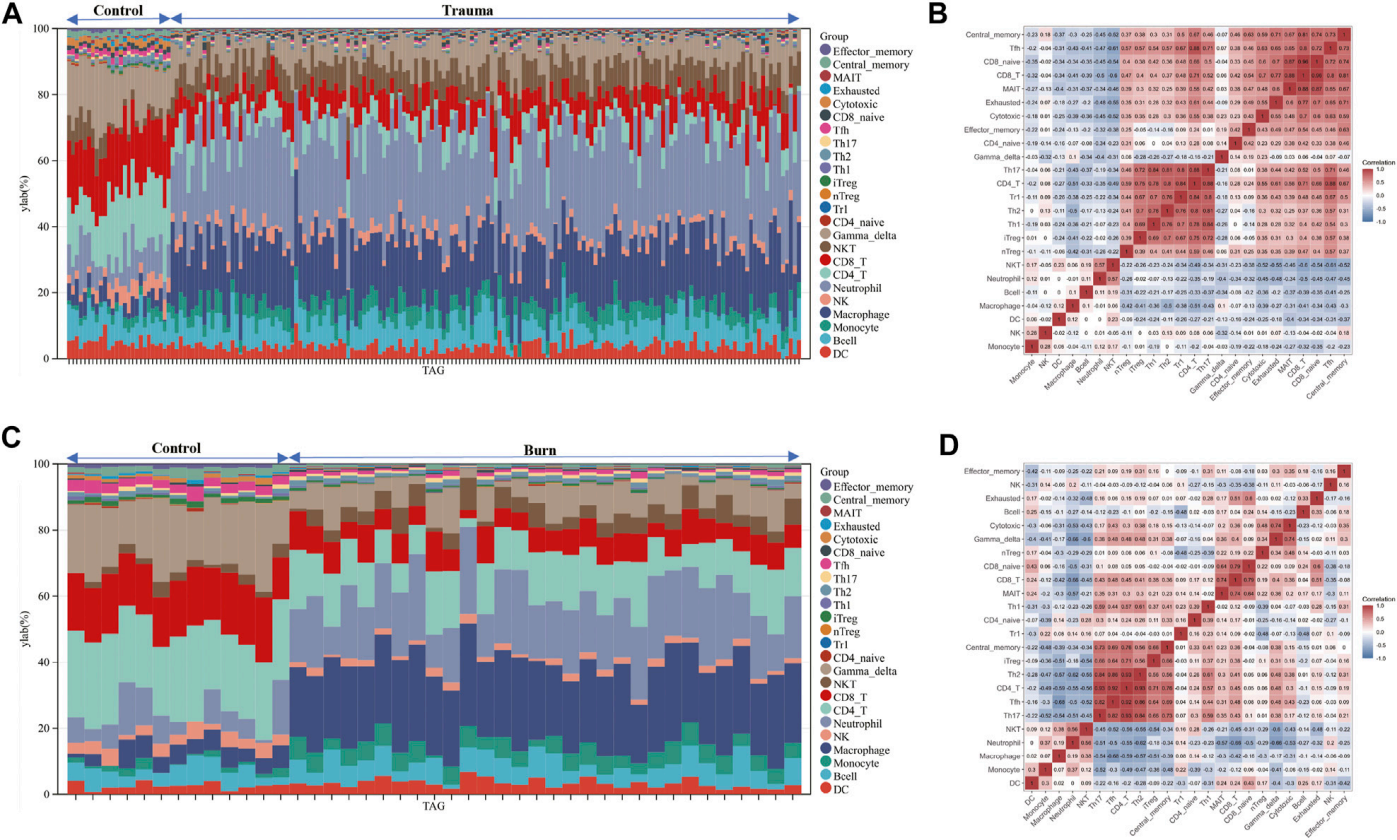
**(A–C)** Stacked bar chart of the immune cell. The different colors of the rectangular bars in the diagram represent different immune cells, and the length represents the proportion of immune cells. **(B–D)** The correlation matrix of immune cell proportions. The numbers in the squares represent the correlation coefficients between the corresponding immune cells.

**FIGURE 6:**
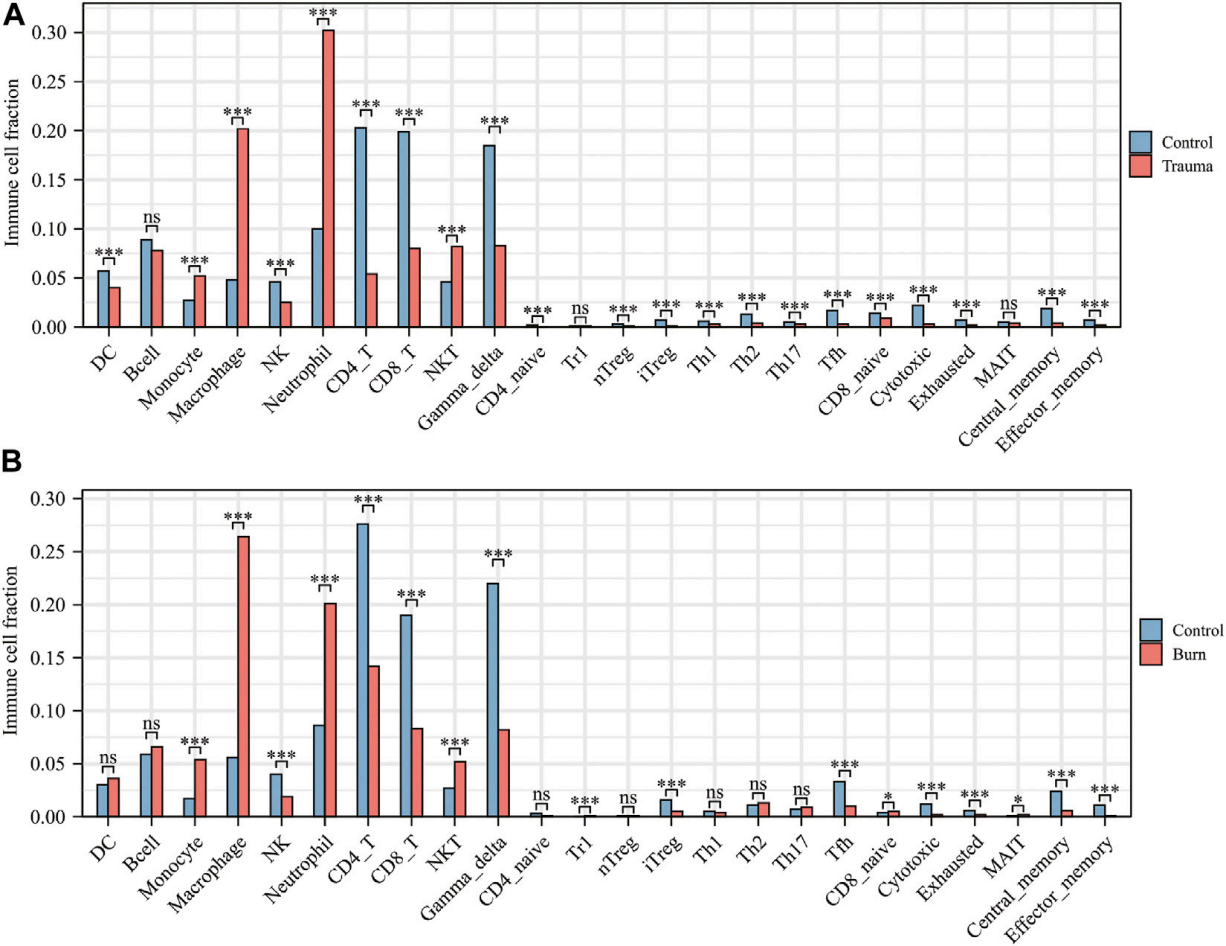
**(A)** Comparison of immune cell fractions between blunt trauma patients and healthy controls. **(B)** Comparison of immune cell fractions between burns patients and healthy controls.

**FIGURE 7:**
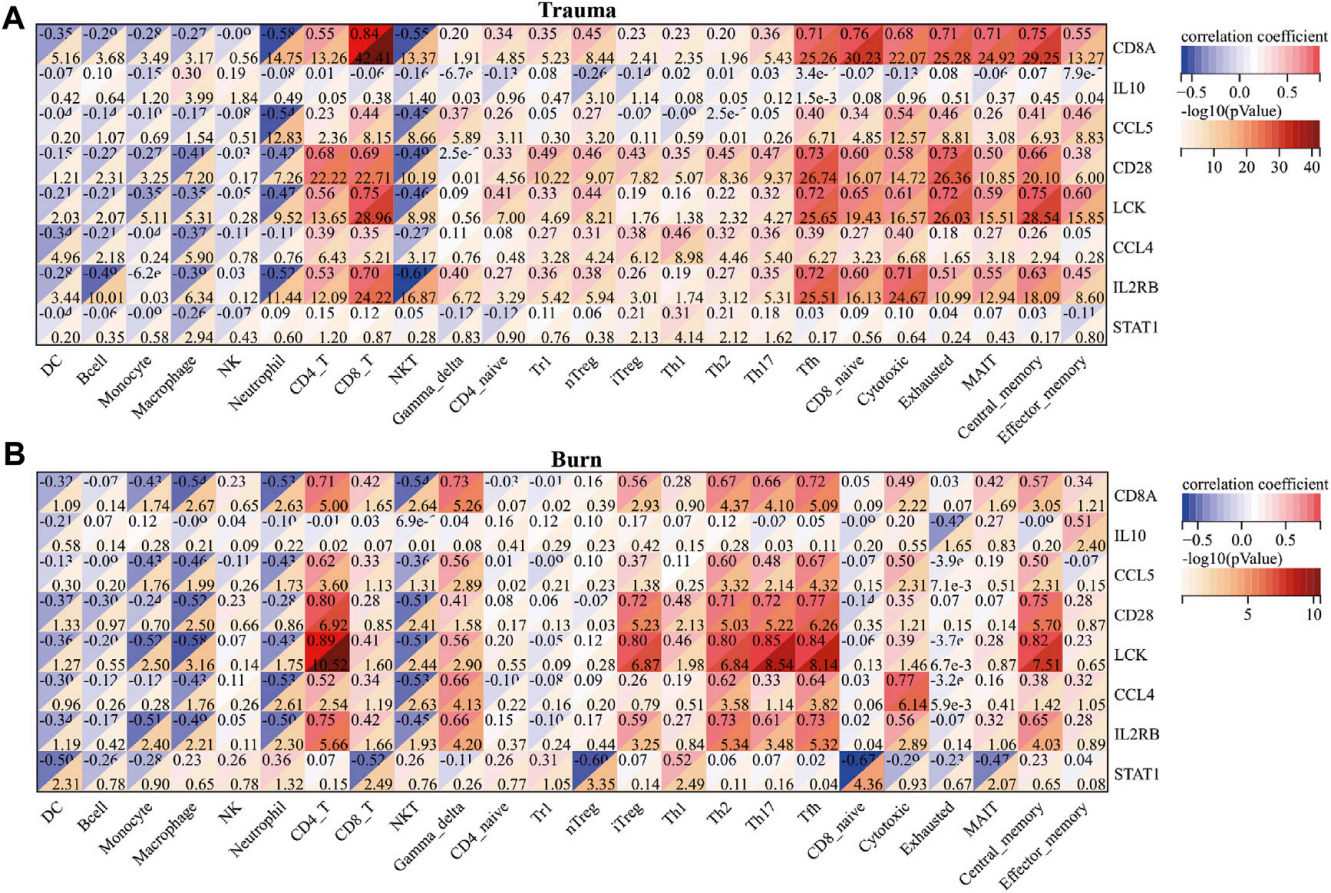
**(A)** Correlations between core immune-related DEGs and immune cell components in blunt trauma patients. **(B)** Correlation between core immune-related DEGs and immune cell components in burns patients.

After evaluating the immune cell composition of blunt trauma and burns patients, we found significant differences in immune cell profiles between diseased and control groups (Figures 5A–C). In blunt trauma and burns patients, Th2, Th17, Tfh, iTreg, and CD4_T were highly positively correlated (Figures 5B–D). In blunt trauma and burns patients, Monocyte, Macrophage, Neutrophil, and NKT were significantly increased, while NK, CD4_T, CD8_T, Gamma_delta, iTreg, Tfh, Cytotoxic, Exhausted, Central_memory, and Effector_memory were significantly decreased (Figures 6A,B). These results suggest a similar profile of immune cell components in blunt trauma and burns patients. In addition, we explored associations between immune-related DEGs and immune cell components in blunt trauma and burns patients. The results showed that the identified immune-related DEGs were more or less significantly associated with simultaneous dysregulation of immune cells in blunt trauma and sepsis patients (Figures 7A,B). For example, NKT was significantly upregulated in both blunt trauma and burns patients. Meanwhile, 7 of 8 immune-related DEGs (CD8A, IL10, CCL5, CD28, LCK, CCL4, and IL2RB) were significantly negatively correlated with NKT in blunt trauma patients (Figure 7A), and 6 of 8 immune-related DEGs (CD8A, CCL5, CD28, LCK, CCL4, and IL2RB) were significantly negatively correlated with NKT in burns patients (Figure 7B).

### Clinical relevance of core immune-related DEGs in blunt trauma and burns

**FIGURE 8:**
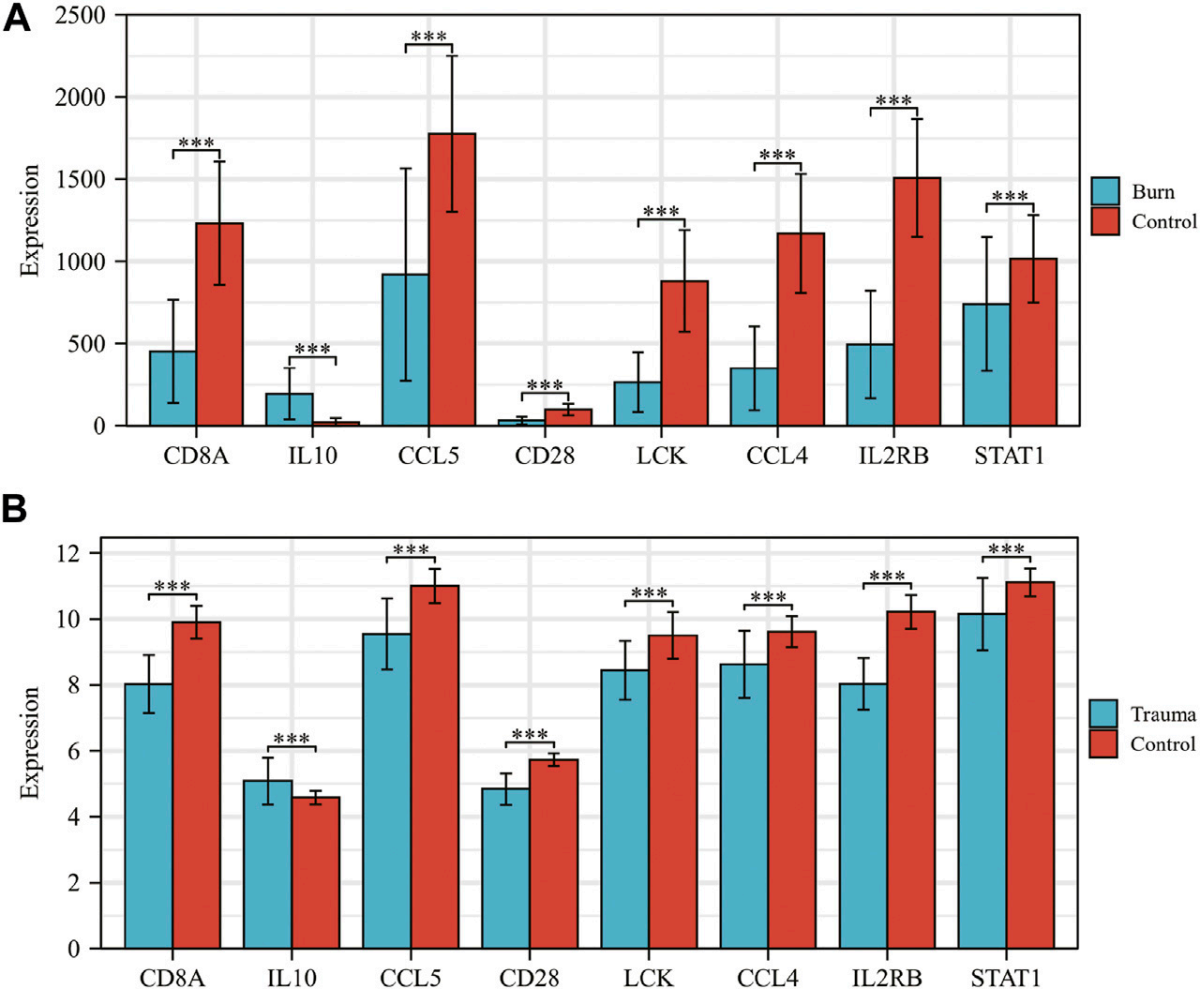
**(A,B)** Expression levels of core immune-related DEGs in GSE36809 and GSE19743.

**FIGURE 9:**
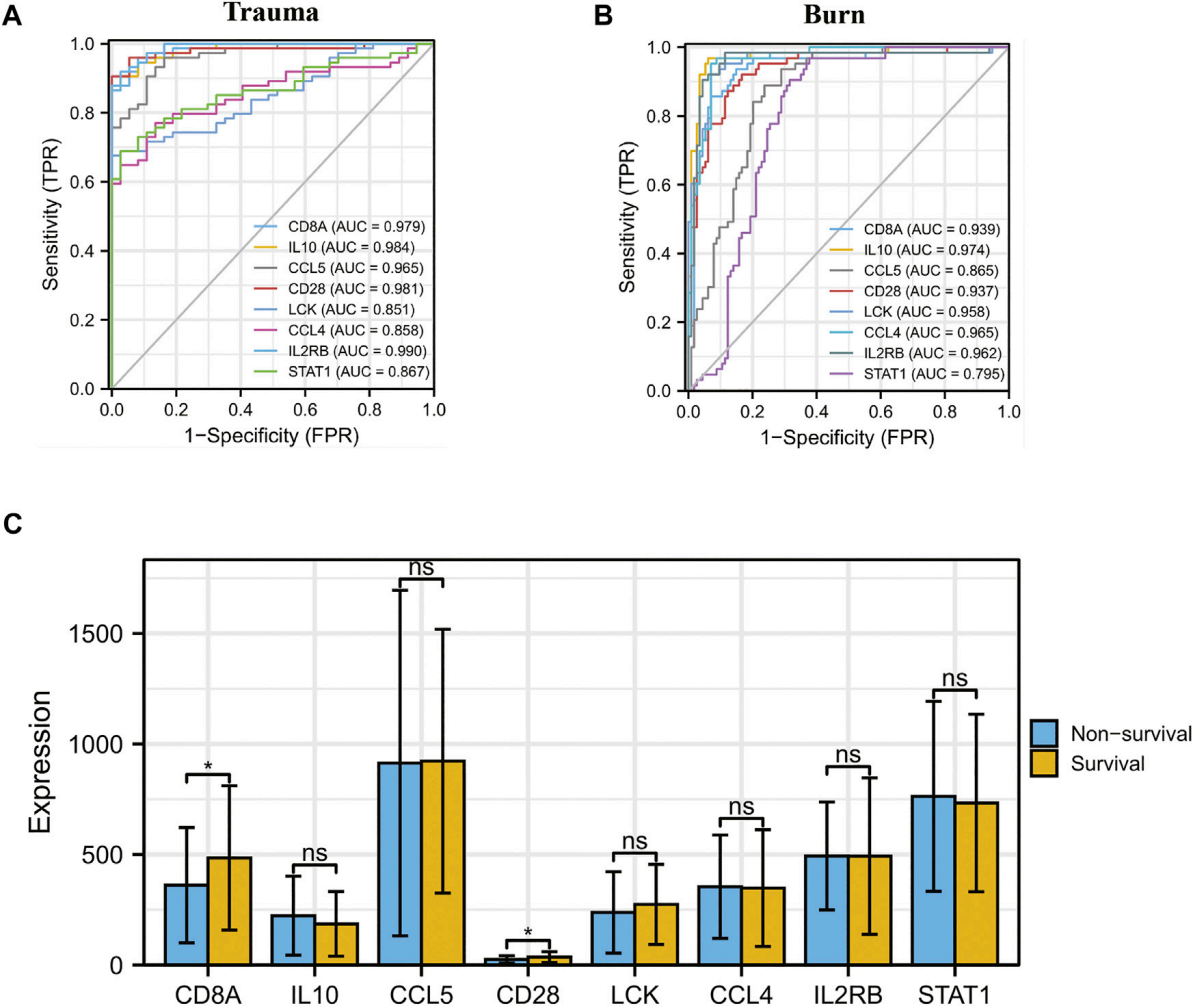
**(A,B)** ROC curve analysis of core immune-related DEGs in blunt trauma and burns. **(C)** Differences in expression of core immune-related DEGs between survival and non-survival of burns patients.

The results of *t*-test analysis showed that 8 immune-related DEGs conformed to the expression trends in the aforementioned blunt trauma and burns datasets (Figures 8A,B). To verify the diagnostic value of immune-related DEGs, we performed receiver operating characteristic curve analysis. The results showed that these genes have good diagnostic potential for both blunt trauma and burns (Figures 9A,B). Since there is survival information for the burns cohort in the GSE19743 dataset, we compared the differences in core immune-related DEGs between the survival and non-survival groups to explore the prognostic potential of these immune-related DEGs for burns patients. The results showed that the expression of CD8A and CD28 was higher in the survival group, indicating that the downregulation of CD8A and CD28 had a worse prognosis (Figure 9C).

## Discussion

In this study, we identified 2103 DEGs and 1752 DEGs in the datasets GSE11375 and GSE77791, respectively. Through Venn diagram calculations, we obtained 117 overlapping immune-related DEGs in GSE11375, GSE77791, and ImmPort databases. We then performed GO and KEGG pathway enrichment analysis. The GO analysis results showed that immune-related DEGs were significantly enriched in protein binding, plasma membrane, inflammatory response, immune response, and cytokine-mediated signaling pathways. These data all indicate that after the body is blunt traumatized and burnsed, the immune regulation is imbalanced, and a series of biochemical reactions occur.

Existing experimental and clinical studies have shown that a few hours after severe burns can produce an extremely dysregulated host inflammatory response (Xiao et al., 2011; Kallinen et al., 2012; Osuka et al., 2014; Sood et al., 2016; Nielson et al., 2017; Greenhalgh, 2019), mainly including cytokine release, elevated protein levels in acute phase, and hypermetabolic state of the body (Jeschke et al., 2008; Rowan et al., 2015). Studies have also shown that after blunt trauma, in addition to activating the immune system, the coagulation and complement systems are also activated (Ganter et al., 2007; Burk et al., 2012; Ekdahl et al., 2016), thereby preventing bleeding and bacterial invasion. In blunt traumatized patients, complement activation products are deposited on the surface of erythrocytes, inhibiting erythrocyte deformability and inhibiting oxygen delivery (Muroya et al., 2014). Inflammatory mediators such as tissue factor, TNF, and C5a, released by damaged tissues and expressed on leukocytes, act as procoagulant factors (Kambas et al., 2008), promoting the generation of thrombin, which causes endothelial cells to release cytokines that promote cell contraction and expression of adhesion molecules (Aird, 2003). The result of these responses is vascular inflammation, perfusion disturbance and tissue hypoxia, which in turn aggravate the thrombo-inflammatory response (Ekdahl et al., 2016; Kral et al., 2016). Damage-associated pattern molecules (DAMPs) and reactive oxygen species (ROS) produced by blunt trauma also induce endothelial cells to produce adhesion molecules to extravasate into damaged tissues (Sun et al., 2013; Vestweber, 2015).

In addition to hypovolemic shock and hypermetabolic response, burns also have important effects on the immune system (Xiao et al., 2011; Finnerty et al., 2013; Seok et al., 2013; Sood et al., 2016). By comparing the number of immune cells between blunt trauma patients and healthy individuals, it was found that the numbers of monocytes, macrophages, neutrophils, and NKT cells were significantly increased in both blunt trauma and burns patients. In response to burns injury, immune cells, including monocytes, macrophages, and neutrophils, are activated within hours to recognize endogenous factors such as DAMPs, activate downstream NF-κB inflammation-related signaling pathways, and promote inflammatory mediators (IL1, IL6, IL8, IL18, and TNF) release, ultimately leading to the development of systemic inflammatory response syndrome (Singer et al., 2016). In addition, antigen presentation by macrophages or killing of invading pathogens by neutrophils, proliferation of T cells, and inhibition of IL2 production can lead to impaired adaptive immune system and enhanced susceptibility (Baker et al., 1979; Antonacci et al., 1984; Wood et al., 1984; Kupper et al., 1985; Schluter et al., 1991; Messingham et al., 2002; Murphy et al., 2004; Miyazaki et al., 2015; Hampson et al., 2017). In addition to the reduced antigen-presenting function of macrophages, the production of IL12 and IL15 is relatively reduced in the early stages of blunt trauma (Stephan et al., 1987; Ayala et al., 1996; Kawasaki et al., 2006; Kawasaki et al., 2009; Hietbrink et al., 2013). Interestingly, there are also studies reporting that IL4 and IL10 can significantly inhibit the antigen presentation of macrophages and the bactericidal activity of NK cells and neutrophils (Donnelly et al., 1991; Fiorentino et al., 1991; Oswald et al., 1992). Our comparison of the associations between immune-related DEGs and immune cell components in blunt trauma and burns patients also found that immune-related DEGs were more or less associated with simultaneous dysregulation of immune cells in blunt trauma patients, for example, NKT in both blunt trauma and burns patients was significantly up-regulated, while IL10 was negatively correlated with the number of NKTs in blunt trauma patients.

We found significant differences in immune cell profiles between diseased and control groups by assessing the number of immune cells in blunt trauma and burns patients. Compared with the control group, the numbers of Th2 and Th17 cells were significantly increased in blunt trauma and burns patients. In addition to impairing the function of the innate immune system, severe burns reduce the number of T lymphocytes that play a dominant role in the adaptive immune system (Heideman and Bengtsson, 1992; Sheridan et al., 1999). Low expression of IL2 and INF-γ and high expression of IL4 and IL10 increase the number of Th2, while suppressing Th1 activity (Schwacha, 2003; Gosain and Gamelli, 2005). The reduction of the Th1 to Th2 ratio is an important factor in suppressing the adaptive immune response (Church et al., 2006). In addition, the ratio of CD4^+^ helper T cells to CD8^+^ suppressor T cells also decreases after severe burns (Burleson et al., 1988). Similarly, burns disrupt the balance between Th17 and regulatory T cells, leading to immune dysregulation. Besides, we have compared the immune cell fraction between the surviving and non-surviving groups for burns. The results showed no significant difference in immune cells (Supplementary Figure S1). We estimate that the reason may be due to the small sample size, with only 8 samples in the non-survival group. However, due to current technical limitations, we cannot distinguish the effects of the cell number and immune-related DEGs expression difference on the immune cell fraction. According to Figure 7B, most of the core immune-related DEGs and Th17 fractions were significantly positively correlated (especially LCK and CD28). We speculate that the expression changes of core immune-related DEGs have a greater impact on immune cell fractions, which further experimental studies are needed to confirm.

In the following *t*-test analysis, we found that eight immune-related DEGs (CD8A, IL10, CCL5, CD28, LCK, CCL4, IL2RB, and STAT1) conformed to the above-mentioned centralized expression trend of the wound and burns dataset. It shows that after the body is injured, the expression of immune-related DEG changes, which in turn mediates the body’s inflammatory response. After severe blunt trauma, a “genetic storm” and functional rearrangement of leukocytes is activated (Lederer et al., 2008; Xiao et al., 2011), mainly through the release of cytokines (IL10), induction of ROS, and mediation of phagocytosis (Munford and Pugin, 2001; Keel and Trentz, 2005; Itagaki et al., 2015; Timmermans et al., 2016; Hazeldine et al., 2017; Seshadri et al., 2017). Notably, the systemic inflammatory response includes not only multiple immune system activation signatures, but also prominent suppressive signatures that evolve within minutes or hours of blunt trauma (Itagaki et al., 2015; Timmermans et al., 2016; Hazeldine et al., 2017).

To verify the diagnostic value of immune-related DEGs, we performed receiver operating characteristic curve analysis. The results showed that these genes have good diagnostic value for both blunt trauma and burns. The expression of CD8A and CD28 was higher in the survival group, indicating that patients with low expression of CD8A and CD28 had poor prognosis. CD28 is an indispensable co-stimulatory molecule for the activation of T cells, which is crucial for the activation of CD8^+^ CTL [(Schwartz, 1992)]. Current studies have shown that CD28 has important effects on T cell function, including transcriptional regulation, post-translational modification, and remodeling of the actin cytoskeleton (Esensten et al., 2016; Zumerle et al., 2017; Porciello et al., 2018), which in turn participates in the regulation of T cell physiological functions, such as the regulation of proliferation signals. Activation, activation of telomerase, and enhancement of T cell migration and homing (Bour-Jordan et al., 2011; Esensten et al., 2016; Zhang and Vignali, 2016). Therefore, downregulation of CD28 is a hallmark of CD8^+^ T cell senescence, and CD28^−^ T cells exhibit marked immunosuppression (Esensten et al., 2016). Importantly, the key regulatory role of CD28 in inducing immune responses has been preliminarily confirmed in animal experiments. After mice were given CD28 antagonists, it was found that CD28 antagonists were able to induce antigen-specific tolerance and prevent autoimmune diseases and organs. The progression of transplant rejection (Lenschow et al., 1992). These results indicate that CD28 is likely to be a promising target for regulating the body’s immune response.

We should acknowledge some limitations of this study. The data of this study are all from public databases and lack the support of clinical data. But based on future trends toward rapid point-of-care testing with limited hands-on time, without the need for specialized laboratories, and the development of statistical methods to analyze gene expression data over time to help address these questions, our study provides some potential targets. Finally, we propose further studies on the regulatory mechanisms and functional roles of dysregulated immune-related DEGs and immune cells to elucidate the temporal association of dysregulated immune-related DEGs and immune cells with MODS in severe disease.

## Conclusion

In conclusion, through a comprehensive analysis of microarray data, our study found that the host immune response is altered after burns and blunt trauma, which may be mediated by specific core immune-related DEGs and immune cells. This study provides potential research targets and directions for further research on the occurrence and development of immune regulation after burns and blunt trauma.

## Data Availability

The datasets presented in this study can be found in online repositories. The names of the repository/repositories and accession number(s) can be found in the article/Supplementary Material.

## Abbreviations

DEGs, differentially expressed genes; PPI, protein‐protein interaction; MODS, organ function disorder syndrome; GEO, Gene Expression Omnibus; TBSA, total burns surface area; GO, Gene ontology; KEGG, Kyoto Encyclopedia of Genes and Genomes; ImmuCellAI, The Immune Cell Abundance Identifier; DAMPs, damage-associated pattern molecules; ROS, reactive oxygen species.
