# Supplementary figures and images for: Genetic and immune crosstalk between severe burns and blunt trauma: A study of transcriptomic data
Source: Front Genet. 2022 Sep 30;13:1038222. doi: 10.3389/fgene.2022.1038222 (PMC9561827; doi:10.3389/fgene.2022.1038222)

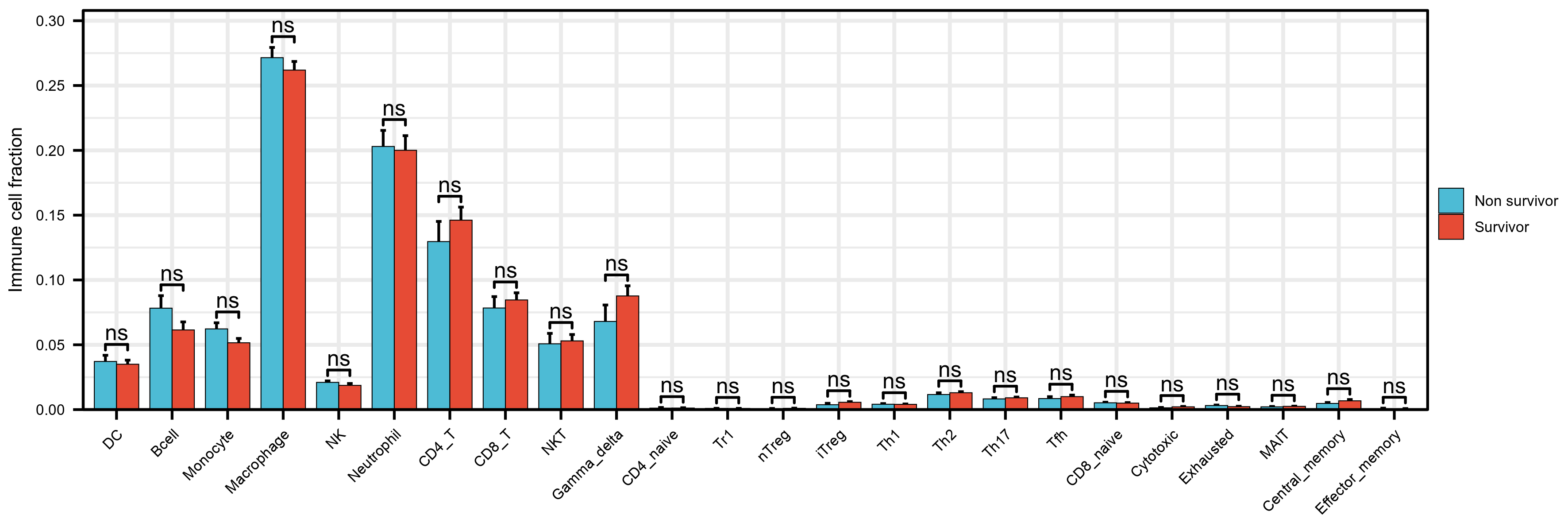

Supplement: Supplementary file 1 [file Image1.tiff]
